# Reclassification of *Chromobacterium violaceum* ATCC 31532 and its quorum biosensor mutant CV026 to *Chromobacterium subtsugae*

**DOI:** 10.1186/s13568-020-01140-1

**Published:** 2020-11-07

**Authors:** Alisha M. Harrison, Scott D. Soby

**Affiliations:** 1 Biomedical Sciences Program, College of Graduate Studies, Midwestern University, 19555 N 59th Avenue, Glendale, AZ 85308 USA; 2 College of Veterinary Medicine, Midwestern University, 19555 N 59th Avenue, Glendale, AZ 85308 USA

**Keywords:** Quorum sensing, Biosensor, *N*-Acyl-homoserine lactone, Molecular phylogeny

## Abstract

The precipitous drop in the cost of genomic sequencing and the concomitant availability of computational methods for comparing genome-level data has made the accurate taxonomic placement of bacteria affordable and relatively rapid. Inaccurate taxonomic placement of bacteria has serious implications in clinical, environmental, and regulatory microbiology, but it can also adversely affect interpretation of research results. The quorum biosensor strain CV026 was derived from an isolate of *Chromobacterium* that was labeled as *C. violaceum* ATCC 31532, and is catalogued by the ATCC under that species name. Nearly 200 papers have been published that use CV026 as an indicator for quorum sensing activity in many Gram negative bacteria, but the inability of *C. violaceum* strains to complement the quorum sensing mutation in CV026 has called the taxonomic placement of the parent strain into question. We used molecular phylogeny and a large number of metabolic and phenotypic characters to demonstrate that *Chromobacterium* strain ATCC 31532 is a member of species *Chromobacterium subtsugae*.

## Key points

- *Chromobacterium* strain CV026 is an important biosensor tool for quorum sensing research but its parent strain ATCC 31532 has been misidentified as *C. violaceum*.
- Molecular phylogeny based on whole genome sequences and phenotypic characteristics indicate that *Chromobacterium* strain ATCC 31532 is most accurately placed in the species *C. subtsugae*.
- *Chromobacterium* strain ATCC 31532 and *C. subtsugae* isolates do not produce HCN, but they do complement the CV026 quorum sensing *cviI* mutation.

## Introduction

The discovery of quorum sensing (QS) as a means of intra- and extra-cellular bacterial communication in 1970 (at the time it was called ‘autoinduction’) (Nealson et al. 1970), and the recognition of QS as a general phenomenon (see for example Whitehead et al. 2001) has resulted in the development and availability of a number of QS biosensors that have been extremely valuable for the discovery and classification of the *N*-acyl homoserine lactones (AHLs) that serve as the primary autoinducers in Gram negative bacteria (Eberhard et al. 1981; McClean et al. 1997). Among the most commonly used biosensors is a mini-*Tn*5 *cviI* mutant of *Chromobacterium* strain ATCC 31532 known as CV026 (McClean et al. 1997; Chernin et al. 1998). Strain CV026 produces an easily-detectable purple phenotype due to the QS-dependent expression of the genes that encode the pigment violacein when complemented with an inducing concentration of medium chain-length HSLs (Chernin et al. 2011; McClean et al. 1997). As of the writing of this manuscript, there are 176 publications indexed in Pubmed since 1997 that use CV026 as a biosensor. However, there has been confusion about the correct taxonomic placement of *Chromobacterium* strain ATCC 31532, which is listed by the ATCC and other national culture collections as *Chromobacterium violaceum*, the genomic sequence of a *Tn*5 mutant of strain ATCC 31532 is catalogued in the GenBank database as *C. subtsugae* CV017 (accession number LKIW01000000), and the vast majority of publications using CV026 refer to it as *C. violaceum*. Because interpretation of QS complementation data can be affected by the taxonomic placement of the strain, it is important to correct the provenance of the parent strain and its QS mutant as an important tool in microbiology.

The species *Chromobacterium violaceum* was first proposed by Bergonzini in 1881 (Gillis and Logan 2015), and ATCC 12472 is the type strain of the species (syn. NCIB 9131, NCTC 9757). In 1982, strain ATCC 31532 was published and deposited in the American Type Culture Collection (ATCC) as *C. violaceum* due to similar phenotypic characteristics associated with *C. violaceum* ATCC 12472^T^ (Wells et al. 1982). *C. subtsugae* was characterized and added to the genus in 2007 (Martin et al. 2007). The genus *Chromobacterium* has further expanded since then with nearly a dozen new species published within the last decade. The absence of affordable and available genomic sequencing tools until the last few years, and the rapid increase in the number of recognized species may have contributed to errors in *Chromobacterium* spp. identification (Kampfer et al. 2018). A recently published study on the reclassification of *C. pseudoviolaceum* LMG3953 showed genomic evidence that *Chromobacterium* strain ATCC 31532 and type strain *C. subtsugae* PRAA4-1^T^ (Martin et al. 2007) were similar enough to warrant a reclassification of strain ATCC 31532 (Kampfer et al. 2018).

In this work we provide genotypic and phenotypic evidence that strain ATCC 31532 is clearly a member of the species *C. subtsugae*.

## Materials and methods

**Fig. 1:**
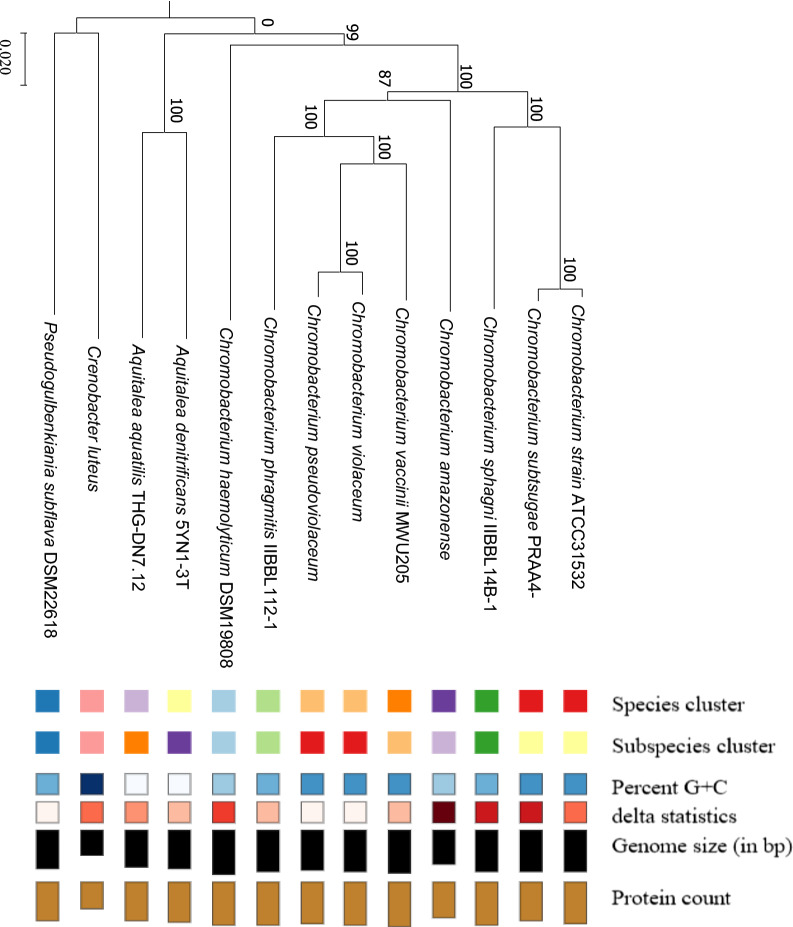
A tree was inferred with FastME 2.1.6.1 from minimum MASH distances. Branch lengths are scaled to Genome BLAST Distance Phylogeny distance formula d5. Pseudo-bootstrap values are shown at the nodes, and are based on 100 replications. The tree has a mean branch support of 98.6%

**Fig. 2:**
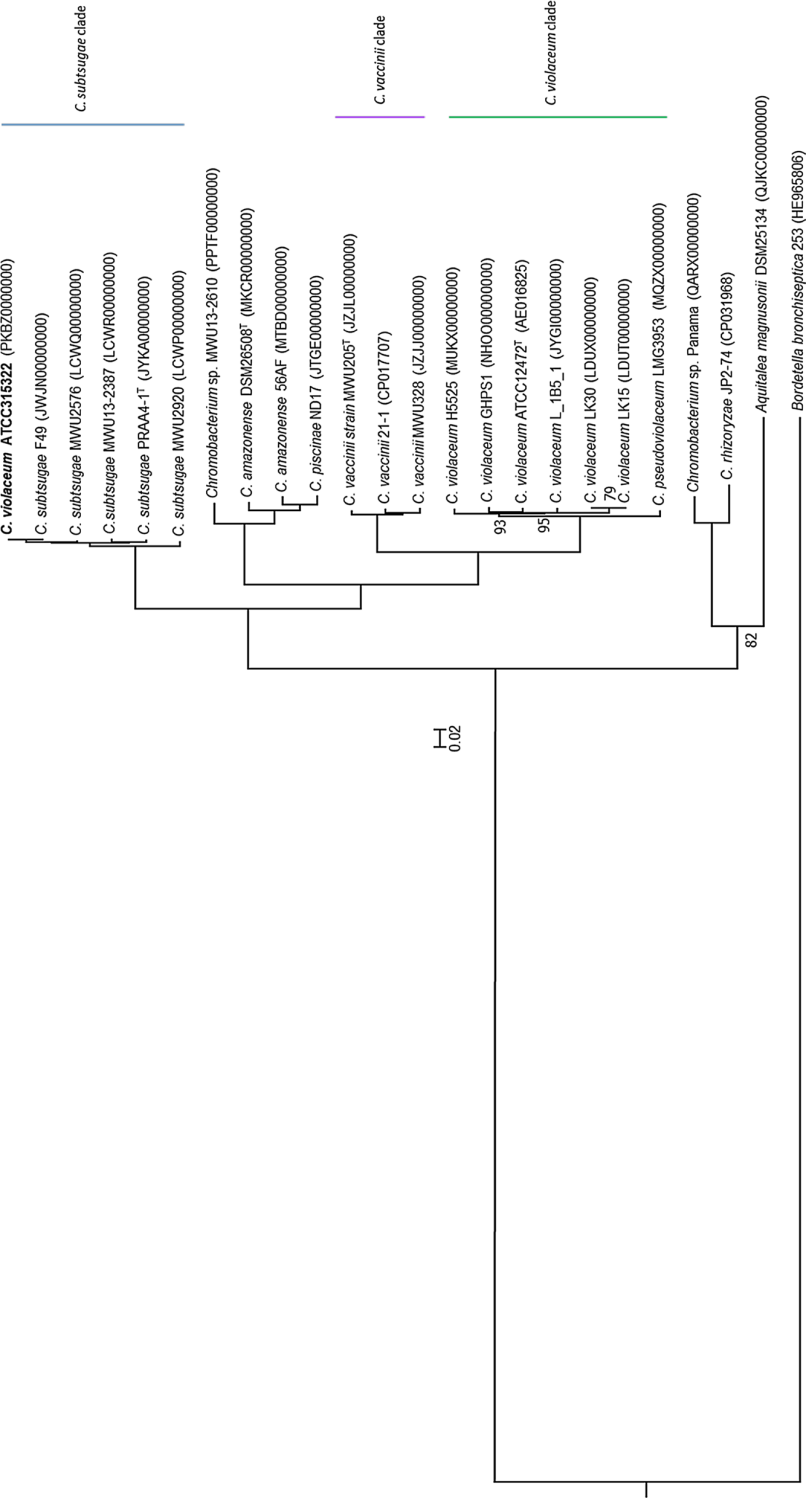
A RAxML phylogenetic tree was constructed in PATRIC using 100 aligned single copy amino acid and nucleotide sequences from a defined number of PGFams. Only bootstrap values less than 100 are shown at the nodes. Accession numbers for genome sequences are shown in parentheses. The isolate known as *C. violaceum* ATCC315322 is unambiguously located in the *C. subtsugae* clade, rather than the *C. violaceum* clade. The β-proteobacteria *Aquitalea magnusonii* and *Bordetella bronchiseptica* serve as outgroups for the family and phylum. The tree is rooted on the *B. bronchiseptica* branch. The scale bar equals 0.02 substitutions

To correctly place strain ATCC 31532 within the genus, we constructed a *Chromobacterium* spp. phylogenetic tree using TYGS (https://tygs.dsmz.de/) with default settings to confirm that *Chromobacterium* strain ATCC 31532 clusters with *C. subtsugae* PRAA4-1^T^ rather than with *C. violaceum* ATCC 12472^T^ the type isolate of the original identification (Fig. 1). This tree is based on the Genome BLAST Distance Phylogeny approach using FastME (Meier-Kolthoff et al. 2019). A second tree was generated with the Codon Trees pipeline in PATRIC version 3.5.43 (https://www.patricbrc.org/) using default settings (Wattam et al. 2017) (Fig. 2) with all of the currently-available *Chromobacterium* spp. genome sequences. Amino acid and nucleotide sequences from a defined number of PGFams (Davis et al. 2016) were randomly selected from a list of single copy genes to build an alignment. A tree was then generated from the differences within the sequences. Protein sequences were aligned using MUSCLE (Edgar et al. 2004), and nucleotide coding gene sequences were aligned using the Codon_align function of BioPython (Cock et al. 2009). No deletions or duplications were allowed in the alignment. One hundred of a possible 423 single-copy coding sequences were used, resulting in alignment of 45,762 amino acids and 137,286 nucleotides. A concatenated alignment of all proteins and nucleotides was used to generate a partitions file for RAxML version 8.2.11 (Stamatakis et al. 2014). Support values were calculated using 100 rounds of the ‘Rapid’ bootstrapping function of RAxML (Stamatakis et al. 2008). Twenty-two *Chromobacterium* spp. genomic sequences were used to compile the tree from genomic sequences of the family *Chromobacteriaceae* with the β-proteobacteria *Aquitalea magnusonii* and *Bordetella bronchiseptica* as outgroups. Additional overall genome relatedness indices (OGRI) comparisons based on variations of Average Nucleotide Identity (ANIb and orthoANIu) were performed using online tools available through JSpeciesWS (http://jspecies.ribohost.com/jspeciesws/) (Richter et al. 2015), digital DNA-DNA hybridization (dDDH) with TYGS and GGDC 2.1 (http://ggdc.dsmz.de/) (Meier-Kolthoff et al. 2013), and EZBioCloud (ezhttps://www.ezbiocloud.net/tools/ani) (Yoon et al. 2017) (Table 1).

**Table 1:** Overall genome relatedness indices (OGRI) comparisons of *Chromobacterium* strain ATCC 31532 with other *Chromobacterium* spp.

Strain	dDDH	orthoANIu	ANIb	% G+C
*Chromobacterium* strain ATCC 31532	*	*	*	64.71
*C. subtsugae* PRAA4-1^T^	93.9	99.28	99.11	64.78
*C. subtsugae* F49	100	99.97	99.94	64.79
*C. subtsugae* MWU2576	95.3	99.42	99.32	64.78
*C. subtsugae* MWU3525	95.0	99.39	99.27	64.86
*C. subtsugae* MWU12-2387	94.0	99.26	99.08	64.82
*C. subtsugae* MWU2920	87.6	98.62	98.33	64.89
*C. sphagni IIBBL 14B-1*^T^	32.9	87.23	86.49	63.42
*C. vaccinii* MWU205^T^	30.0	85.68	84.86	64.35
*C. violaceum* ATCC 12472^T^	30.2	85.78	84.93	64.83
*C. amazonense* DSM 26508^T^	28.0	84.54	83.64	62.61
*C. phragmitis IIBBL 112-1*^T^	28.8	84.83	83.84	63.91
*C. haemolyticum DSM 19808*^T^	23.4	79.88	78.54	62.63

*Chromobacterium subtsugae* strains labelled with ‘MWU’ were isolated from wild and cultivated cranberry bogs and along with *C. subtsugae* F49 are used to illustrate relatedness of *Chromobacterium* strain ATCC 31532 across a range of species members. dDDH was calculated using the d5 (algorithm 2) of both the TYGS and GGDC online tools (http://ggdc.dsmz.de/home.php). OrthoANIu and % G+C was calculated using the ezbiocloud online ANI tool (https://www.ezbiocloud.net/tools/ani), which compares two genome sequences using the OrthoANIu algorithm. ANIb was calculated using the online tool JSpeciesWS (http://jspecies.ribohost.com/jspeciesws/). Strains below the seveth row in the table fall below established criteria for species classification

Phenotypic and metabolic tests comparing isolates *C. violaceum* ATCC 12472^T^, *C. subtsugae* ATCC 31532 and *C. subtsugae* PRAA4-1^T^ were conducted using API 20 NE test strips (BioMerieux) and Biolog GEN III plates according to the manufacturers’ instructions. Altogether we generated comparative data from over 100 different metabolic and phenotypic tests. Production of hydrogen cyanide (HCN), and AHL complementation tests were also conducted to further increase the reliability of taxonomic assignment of strain ATCC 31532 to either the *C. violaceum* or the *C. subtsugae* subclades. HCN quantification was performed as previously reported (Ebadzadsahrai et al. 2020). Cells were grown to late log phase at 26 °C in King’s Medium B broth and pelleted by centrifugation. Cell-free supernatant pH was adjusted to pH 11 by the addition of 50 µl 1N NaOH per ml supernatant, and measured directly using an ion-specific electrode (Lazar Research Laboratories) as previously described (Zlosnik and Williams 2004). Measurements were converted to ppm with a standard curve.

AHL complementation of the QS mutant CV026 was determined by growing the biosensor strain adjacent to strain ATCC 31532, *C. subtsugae* PRAA4-1^T^, or *C. violaceum* ATCC 12472^T^ on King’s Medium B agar plates for 24 h at room temperature. The biosensor strain does not produce its own AHL, therefore it does not produce the purple pigment violacein. In this assay, complementation of the *cviI* mutation occurs by diffusion of exogenously applied or biologically produced AHLs through the agar to the biosensor strain in sufficient concentration to trigger the QS response, resulting in the production of violacein.

## Results

### Phylogenetic evidence

Strain ATCC 31532 is located within the *C. subtsugae* clade of both GDBP and RAxML *Chromobacterium* spp. genomic phylogenetic trees, indicating its close relationship to that species, and not within the *C. violaceum* or other clades (Figs. 1 and 2). All of the other clades were internally consistent, and had strong bootstrap support in both trees. OGRI comparisons using three different algorithms, ANIb, OrthoANIu, and dDDH confirm the close relatedness of *Chromobacterium* strain ATCC 31532 to *C. subtsugae*, particularly to strain F49, but also well within the defining limits of 95–96% for ANI, 70% for dDDH, and 1% for G+C with all of the *C. subtsugae* strains in our collection, and well outside the limits of *C. violaceum* ATCC 12472 and the other Chromobacterium species (Table 1).

### Phenotypic evidence

Most metabolic and phenotypic characteristics examined were shared among all three isolates, as would be expected for members of the same genus, but 22 characteristics were the same for strain ATCC 31532 and *C. subtsugae* PRAA4-1^T^ that differed from *C. violaceum* ATCC 12472^T^ (Table 2). There were no metabolic differences between strain ATCC 31532 and *C. subtsugae* PRAA4-1^T^. The type isolate *C. violaceum* ATCC 12472 reduced nitrate, hydrolyzed aesculin, and utilized d-mannose, sucrose, adipic acid, pectin, and glucuronamide whereas strain ATCC 31532 and ^T^*C. subtsugae* PRAA4-1^T^ did not. Strain ATCC 31532 and *C. subtsugae* PRAA4-1^T^ utilized dextrin, a-d-lactose, d-salicin, d-galactose, 3-methyl glucose, l-fucose, d-arabitol, *myo*-inositol, l-pyroglutamic acid, citrc acid, and *N*-acetyl-d-galactosamine, whereas *C. violaceum* ATCC 12472^T^ did not. HCN is produced by both *C. violaceum* (Michaels and Corpe 1965) and *C. vaccinii* (Voing et al. 2015; Ebadzadsahrai et al. 2020), but has not been reported from *C. subtsugae*. *C. violaceum* ATCC 12472^T^ supernatants contained 81 ppm HCN, *C. subtsugae* PRAA4-1^T^, MWU 12-2387, and strain ATCC 31532 supernatants contained about 10 ppm, which is the baseline level of the growth medium (Fig. 2). CV026, the *cviI* mutant of strain ATCC 31532, was complemented by its parent strain ATCC 31532 as well as by *C. subtsugae* PRAA4-1^T^, and *C. subtsugae* MWU 12-2387 (Voing et al. 2017), but not by *C. violaceum* ATCC 12472^T^, which indicates that strain ATCC 31532, *C. subtsugae* PRAA4-1^T^, and *C. subtsugae* MWU 12-2387 produce *N*-acyl-homoserine lactones (AHLs) that are recognized by CV026, but the AHLs produced by *C. violaceum* ATCC 12472 ^T^ are not.

**Table 2:** Phenotypic characteristics of (1) *C. violaceum* ATCC 12472^T^, (2) *Chromobacterium* strain ATCC 31532, and (3) *C. subtsugae* PRAA4-1^T^

Characteristics	1	2	3
Nitrate reduction	+	−	−
Aesculin hydrolysis	+	−	−
pH growth ≥ 5	−	+	+
Utilization of			
d-Mannose	+	−	−
Sucrose	+	−	−
Adipic acid	+	−	−
Pectin	+	−	−
Glucuronamide	+	−	−
Dextrin	−	+	+
a-d-Lactose	−	+	+
d-Salicin	−	+	+
d-Galactose	−	+	+
3-Methyl glucose	−	+	+
l-Fucose	−	+	+
d-Arabitol	−	+	+
Myo-inositol	−	+	+
l-Pyroglutamic acid	−	+	+
Citric acid	−	+	+
*N*-acetyl-d-galactosamine	−	+	+
Cyanide production	+	−	−
CV026 complementation	−	+	+

## Discussion

**Fig. 3:**
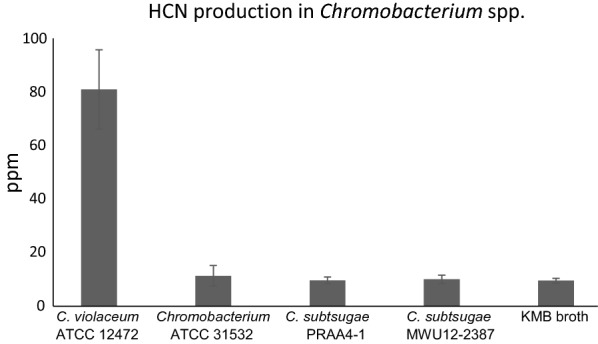
Hydrogen cyanide production in *Chromobacterium* app. isolates. *C. violaceum* produces about 70 ppm HCN. *C. subtsugae* PRAA4-1, MWU 12-2387, and *Chromobacterium* strain ATCC 31532 do not produce detectable amounts of HCN above background

Taxonomic placement of *Chromobacterium* strain ATCC 31532, the parent strain of the widely-used QS biosensor CV026, is critical for the correct interpretation of complementation experiments because *C. violaceum* and *C. subtsugae* use different autoinducers (Stauff and Bassler 2011; Rekha et al. 2011; McClean et al. 1997). We have used both genotypic and phenotypic evidence to show that the parent strain of the biosensor CV026 has been misidentified and deposited in the American Type Tissue Collection (ATCC) and other national repositories as *C. violaceum* because it was deposited long before *C. subtsugae* had been described and included in validated prokaryote species lists. Several complementary phylogenetic and genotypic analyses confirm the placement of *Chromobacterium* strain ATCC 31532 in the species *C. subtsugae*, including very different types of OGRI algorithms (Table 1). A large number of metabolic and other phenotypic tests also indicate that strain ATCC 31532 is a member of the species *C. subtsugae*, and not *C. violaceum* (Table 2). The primary AHL produced by *C. subtsugae* PRAA4-1^T^ is *N*-hexanoyl-l-homoserine lactone (C6-HSL) (McClean 1997) whereas the AHL produced by *C. violacein* ATCC 12472^T^ is *N*-decanoyl-l-homoserine lactone (C10-HSL) (Morohoshi et al. 2008). Further evidence is the production of HCN by *C. violaceum* but not by *C. subtsugae* strains, including ATCC 31532 (Fig. 3). Based on both genotypic and phenotypic characterization, it is clear that *Chromobacterium* strain ATCC 31532, and therefore the widely used biosensor strain CV026, have been misidentified as *C. violaceum*, and should be re-designated *C. subtsugae* ATCC 31532.

## Data Availability

All data used in the phylogenetic analysis are publicly available in GenBank. Microbial strains are publicly available, or in the case of *C. subtsugae* lab strains may be requested from the authors.
